# Heart Palpitation From Traditional and Modern Medicine Perspectives

**DOI:** 10.5812/ircmj.14301

**Published:** 2014-02-05

**Authors:** Tabassom Ershadifar, Bagher Minaiee, Manouchehr Gharooni, Mohammad Mahdi Isfahani, Alireza Nikbakht Nasrabadi, Esmaiel Nazem, Ashraf Aldin Gousheguir, Davod Kazemi Saleh

**Affiliations:** 1 Department of Iranian Traditional Medicine, School of Iranian Traditional Medicine, Iran University of Medical Sciences, Tehran, IR Iran; 2 Department of Histology, School of Medicine, Tehran University of Medical Sciences, Tehran, IR Iran; 3 Department of Cardiology, Tehran University of Medical Sciences, Tehran, IR Iran; 4 Department of Iranian Traditional Medicine, School of Iranian Traditional Medicine, Tehran University of Medical Sciences, Tehran, IR Iran; 5 Research Center of Quran, Hadith and Medicine, Tehran University of Medical Sciences, Tehran, IR Iran; 6 School of Nursing and Midwifery, Tehran University of Medical Sciences, Tehran, IR Iran

**Keywords:** Palpitation, Medicine, Traditional, Iran, Temperament

## Abstract

**Background:**

Palpitation is a sign of a disease and is very common in general population. For this purpose we decided to explain it in this study.

**Objectives:**

The purpose of this study was to describe the palpitation in both modern and traditional medicine aspect. It may help us to diagnose and cure better because the traditional medicine view is holistic and different from modern medicine.

**Materials and Methods:**

We addressed some descriptions to the articles of traditional medicine subjects which have published recently. Palpitation in modern medicine was extracted from medical books such as Braunwald, Harrison and Guyton physiology and some related articles obtained from authentic journals in PubMed and Ovid and Google scholar between1990 to 2013.

**Results:**

According to modern medicine, there are many causes for palpitation and in some cases it is cured symptomatically. In traditional medicine view, palpitation has been explained completely and many causes have been described. Its aspect is holistic and it cures causatively. The traditional medicine scientists evaluated the body based on Humors and temperament. Temperament can be changed to dis-temperament in diseases. Humors are divided in 4 items: sanguine, humid or phlegm, melancholy and bile. Palpitation is a disease, it is heart vibration and is caused by an abnormal substance in the heart itself or its membrane or other adjacent organs that would result in the heart suffering.

**Conclusions:**

Our data of this article suggests that causes of palpitation in the aspect of traditional medicine are completely different from modern medicine. It can help us to approach and treat this symptom better and with lower side effects than chemical drugs. According to this article we are able to detect a new approach in palpitation.

## 1. Background

Palpitations are a common presentation in general practice and a frequent reason for cardiology referrals, associated with marked disability (1). However, palpitations are often benign, Less than half of patients with palpitations suffer from an arrhythmia and not every identified arrhythmia is of clinical or prognostic significance, there is also a high incidence of anxiety disorders among patients presenting with palpitations. The consideration of this topic had the potential to connect and integrate Iranian traditional medicine to current medicine. It opens new frontiers for the physiopathology of modern medicine palpitation according to traditional medicine.

In Traditional Medicine, heart diseases are being dealt with a different approach from the modern medicine. The ancient scientists have categorized palpitation according to its causes, for example, in old time, the scientists would pay attention to the causes of illness mostly based on hot, cold, wet or moist, dry (2) and the combination of hot and wet, hot and dry, cold and wet and cold and dry dis- temperament. They paid attention to a disease as well. If it is caused by cold dis-temperament, it may not be accompanied by high heart rate and we may experience slow pulse rate in the patient. In opposite we can feel the patient’s pulse increases if the body heat is raised or if the temperament is hot. If they would see the symptoms of hot temperament in a patient, they would use measures that went against the hotness effects and prescribed medicines to cool down the body or the heart. For instance, in hot palpitation with symptoms like fast pulse, thirst, weight loss and need for cold air, they used to prescribe medicines or adopt measures to cool down the body hotness. In this paper in addition to the traditional medicine we put modern medical views on palpitation besides the herbs which have been used in palpitation according to Avicenna and Razi aspects too.

## 2. Objectives

This article aimed to determine the traditional scientist aspects regarding the causes, signs and symptoms of palpitation and also useful herbal drugs for treatment based on traditional medicine books. Therefore, we decided to study this disease not only from traditional medicine point of view due to its importance in the current society but also of modern medicine.

## 3. Materials and Methods

This is a review article study. Our keywords exist in PubMed and ITM. In this review study specific data related to the subject among all referral ITM texts was extracted from original and authentic medical ancient books such as Hakim Azam Khan, Ibn e Sina, Hakim Arzani and other famous scientists. Palpitation has been selected from the traditional medicine (ITM) books of famous scientists and we brought the etiology, signs and symptoms here, and in the meantime the single herbal medicines shown in table from 500 ones in Avicenna’s book were selected in such way that those herbs effective on palpitation has been reported. Similar references were made to the Razi’s Alhavi, in which few common single herbs were determined, also some are beneficial for heart and palpitation have been brought from Exir and Makhzan-al-Advieh which are authentic ITM books.

For some more explanations, some related ITM articles of Iranian educated students which were published in authentic journals have been inserted here. Their subjects and their journals had been known, then they detected easily. Also references to modern medicine were made from sources such as Braunwald and Harrison, Guyton and Hall (textbook of medical physiology) and using journal sources also contributed to this survey for palpitation. References to modern articles were searched by: PubMed, Ovid and Google scholar.

 First, PubMed, based on keyword- palpitation was searched. Detail searched of palpitation in journal fields were: human and free full text available and published in the last 10 years from 2004 to 2013. Then 20 articles from 106 were found, after reviewing these 20 articles only one was related to this subject: [Experience in syndrome differentiation and treatment of palpitation] seemed related to the subject, but after opening, although it was written free full text, it was not opened.

Another site was Ovid. Palpitation as our keyword was searched in published last 13 years (from 1990 to 2013).Then only 5 text were found .One was related to this subject. Searching in Google scholar by palpitation keyword, 3 additional articles related to this subject were found. At enjambment, to bring those articles in endnote, they were searched in Google scholar and were sent to the endnote of this text. 

## 4. Results

In Traditional medicine, heart is the most important organ in the body (3), which its superiority of directing as a chief in the body is more important than other organs and its diseases are being dealt with a different approach from the modern medicine. In this medicine with a background of thousands years, the Palpitation, a kind of heart diseases was well described. In another word for example in the old medical books from 3rd to 12th Hijrah century, we notice that the heart and its diseases are very well mentioned and also through later centuries due to more discovery of heart diseases. In this paper we try to explain palpitation as one of the heart disease. Of course they have categorized palpitation according to its causes which reveal different symptoms. So they are different one from the other according to the cause. For instance, if it is caused by body coldness, it may not be accompanied by increased rate and in contrary to that we may experience slow pulse in the patient. This is while we can feel the patient’s pulse increases if the body heat is raised .Based on this, we may claim that hot palpitation is accompanied by increasing in rate (4)(5)(6). In old time, the scientists would pay attention to the causes of illness and in addition to curing the disease they would also treat its cause as well. For example if they would see the symptoms of hot palpitation in a patient, they would use measures that went against the hotness effects and prescribed medicines to cool down the body or the heart. For some instance, in hot palpitation with symptoms like fast pulse, thirst, weight loss and need for cold air, they used to prescribe medicines or adopt measures to cool down the body hotness first and then they would use treatments to strengthen the heart, so that it wouldn’t become sick again (3).

### 4.1. Describing Palpitation- Modern Medicine

Palpitations are a common presentation in general practice and a frequent reason for cardiology referrals (4) associated with marked disability (1). However, palpitations are often benign, Less than half of patients with palpitations suffer from an arrhythmia and not every identified arrhythmia is of clinical or prognostic significance, there is also a high incidence of anxiety disorders among patients presenting with palpitations (4). In study over 190 patients with palpitations symptoms in the modern medicine the following results were found:

31 % with psychological cause.

16 % with un-identified cause.

10 % with miscellaneous cause.

42 % with cardiac causes (5).

Palpitations refer to a patient's awareness of a forceful, irregular, rapid, pounding or otherwise unusual heart rate. The signs of palpitation range from excessive awareness of normal variations in heart rate to life-threatening heart conditions. Evaluation must determine which individuals only require reassurance and those who need extensive evaluation and treatment (6, 7). Palpitation in the view of Harrison Internal medicine has described as an unusual awareness of the cardiac impulse (8). This impulse could be slow, fast, regular or irregular or just a sense of a hard beat. Of course the difference between knowing the regular and irregular cardiac impulses is that the individual must concentrate on his heart to feel the impulses while in the second place the individual’s thoughts are disrupted (Dorland Medical dictionary (9)).Cardiac impulses are best felt when the patient is resting with minimum external irritants. Sometimes the cardiac pulses are conditional for instance in such situation palpitation is caused in certain position in atrium myxoma or presence of a mass in the chest (8). I t is possible to feel palpitation in the heart, in the chest, in the throat or in the neck. Another symptom of palpitation is a flip-flopping, rapid fluttering in the chest and pounding in the neck (10). In general there are between 60-100 heart beats per minute. People with regular daily exercise or under certain medication may experience less than 55 beat per minute. Usually palpitation is not a serious disease. However if accompanied by other symptoms such as perspiration, faint, alternate headaches, vertigo or chest pain, it is an indications of weak or irregular heart function which must be seriously examined (5).

One of the most common causes of palpitation is panic attacks (11). It is difficult to diagnose between the panic and arrhythmic attacks because in both of them there are the same symptoms such as dyspnea and dizziness. Syncope is also one of the unusual symptoms for panic disorders, it is caused by increasing in sympathetic activities, there might be arrhythmia found in panic attacks but with no specific heart problem, Palpitation can also be witnessed in anxiety. Some of the cardiac causes for palpitation are as follows: premature contraction of atrial and ventricular and post ventricular arrhythmia, mitral prolapsed, atrial fibrillation, aortic regurgitation and atrial myxoma.

Palpitation also is seen in hyperdynamic situations such as increasing body temperature, blood loss (12), coronary artery diseases that are accompanied by catecholamine release, like exercise (Sport) ,Stress, pheochromocytoma , and pregnancy (10, 13).

### 4.2. Miscellaneous Causes

Sudden skeletal muscle contractions of the chest, Thyrotoxicosis, systemic mastocytosis, low blood sugar level, hypoxia, hyperventilation, asthma, medicine consumption such as: beta blockers, anti-arrhythmia, caffeine, cocaine, amphetamines and ethanol (5, 6) and also an Ibuprofen palpitation side effect (14), Asthma medications like albuterol inhalers and some thyroid replacement medications are also known to cause side effects that include palpitations. The use of certain anti-depressants may also cause various symptoms like nausea, indigestion, constipation and heart palpitations (11, 13).

### 4.3. Describing Palpitation-Traditional Medicine

In traditional medicine, heart is the first body organ to start its function and is the last to stop (15). Heart is an organ that is affected by anger, exhilaration and bashfulness .An animal with bigger heart and more hot tempered is more courageous (3, 16). Often, heart is located at the right side, instead of left side .In the past scholars used to categorize diseases based on their causes (3). As it was mentioned before, for example due to predominance of coldness over the heart, they used to call it cold dis-temperament, or as a result of hot predominance over the heart, they would call it hot mal- temperament of the heart. They also believed that a series of heart diseases were related to other organs. For example, in stomach, liver, lung, digestive system, uterus and even diaphragm diseases, heart can be involved and become sick (17).

According to Avicenna (18), palpitation is a heart vibration and is caused by a substance in the heart itself or its membrane or other adjacent organs that would result in the heart suffering. This substance may come from a humor, a simple dis-temperament (19), an inflammatory situation, a weird reason like an infection or a great fear. This humor may be sanguine, humid or phlegm, melancholy or bile .If this is caused by a simple dis-temperament, and affects the whole body situation, it will result in weakness. When weakness affects the heart and continues then it leads to anxiety, therefore, the reaction by the heart to repel this problem may be in the form of palpitation. If palpitation extends to its extreme then it would be transformed into syncope (18). Ali Ibn e Abbass e Ahvazi describes palpitation in his book, Kamel al Sanaeh as follows (17):

If there is humidity within the heart membrane, the heart cannot expand or contract normally and as a result, the individual may feel distraction and vibration in his heart. However, inflammation can be another cause as well.

Based on the definition given by Ibn Nafis Gharshi in his book Almoojez (16) the heart distraction is caused by repelling the distressing agents. Davood Antaki (20) explains in his book Boghyat ol Mohtaj: Khafaghaan is a vibratory movement in heart which is caused by an inappropriate substance in it. This can be a result of a long sickness in which the patient loses his strength, mal-nutrition or drinking, over bleeding, an infectious humor or a stomach malfunction. Hakim Mohammad Arzani describes palpitation in his book: Tebbe Akbari, if the cardiac beats as a vibratory movement in the heart will hurt the heart (21). 

Hakim Mohammad Azam Khan-e-Chashti (3) views palpitation in his book, titled: Exir-e-Azam as a heart mal function that is caused by anything distressing the heart. This hurtful cause could be within the heart or in its membrane or in the adjacent organs like brain, stomach, liver, lung, uterus, intestine or in the whole body that would contribute to distressing the heart .This sometimes comes from humor or non-humor substance, or simple dis-temperament or sometimes from dissociation or blockage, infection, or weakness of the heart. If it is the humor, sanguine, bile, phlegm or melancholy which may block arteries however bile is rarely evident. The phlegmatic wet humor can be accumulated in the heart membrane or it may be due to melancholic humor that would block the cardiac arteries. Whatever of simple dis-temperament or any dis-temperament that dominates the heart would bring around weakness and any weakness of the heart in return may be the cause for the heart palpitation which repels the distress, provided that the heart is yet strong enough. If palpitation extends to its extreme, it would transform in to syncope (18). Then we can explain the causes of palpitation in traditional medicine view as below:

Dis-temperaments, dissociation, obstruction in blood vessels and lung arteries and any joint organs, such as stomach, brain, uterus, lung, intestines and liver disease are the causes of palpitation. Obstruction may be caused by inflammation of adjacent organs, animal bites and poisons, worms in the digestive system especially when they move to upper parts and their wastes move closer to heart. Also we can point out to the strength of the heart sense that could cause palpitation by any little distress. This palpitation could be as a result of exercising, excessive ejaculation, hemorrhoid bleeding, mal nutrition or bad drinking or even desire to eat mod (3). Palpitation as a result of participatory diseases may be caused by soft or hard inflammation of the heart membrane and or jointly with stomach. For example there is humor or a thick or burning substance or rashes in upper part of stomach that can cause palpitation. Also mal digestion of food in stomach can be cause of palpitation, even series of movements in upper part of stomach can have symptoms similar to palpitation (3, 22).Other joint organs which can cause palpitation is lung (18). Anytime palpitation becomes chronic, it will cause the heart weakness which in return may transform syncope and after a while it may end in sudden death (3).

### 4.4. Diagnosis

In order to diagnose different types of palpitation or khafaghan, we may take the patients pulse to find out if there is any change in the pulse regarding its altitude, speed or if it is felt under four fingers. In certain cases of palpitation, the voice can be heard while lying down on his left side and resting on his ear (3). We can take advantage of these dis-temperaments in diagnosing different types of palpitations. For example, in hot and wet dis-temperament the symptoms are as follows: heat and inflammation out of palpitation time and body benefit of intercourse and the symptoms like urine high density, swelling, blood vessels, heaviness and upset in body organs, motion retardation, mouth sweetness, excessive perspiration and loss of appetite. However, in bile dis-temperament we may notice excessive inflammation, sleeplessness (insomnia), restlessness, excessive thirst, mouth and tongue bitterness, yellowish face, headache, suffering the weather heat or enjoying the cold , and the signs in which the speed and the hardness of the pulse (3).

The symptoms for melancholic dis-temperament or black-bile temperament (23) are panic and sadness, hallucination and bad thoughts, semi- depression, nightmares and hardness of pulse. If the pulse is softly felt under fingers and the urine color is white and there are symptoms of wet dis-temperament, the patient feels like his heart is floating in the water and will be overwhelmed by syncope and dyspnea. This type of dis-temperament is phlegmatic, meaning that coldness and humidity dominate over the heart .We stop mentioning further other causes of palpitation such as participatory, clogging and inflammation (3).

### 4.5. General Treatment of Palpitation

In traditional medicine treatment of diseases are addressed in a wide spectrum. In this treatment we can use different means such as fragrances (aromas), ointments, cutting artery or phlebotomy, cupping, and dieting in addition to medication. For instance in the hot palpitation we table advantage of smelling rose water, camphor and sandal wood as well as using ointments that contain saffron and dianthus caryomphyllus (clove). It is noteworthy to prescribe soft foods (quick digest foods) and foods that would support or strengthen the heart .In this food prescription we should take note that the patient does not take flesh or if he does, he should take chicken meat. Other ways of treating palpitation are as follows: 

Rose water soaked raisin or raisin soaked in mix rose water and pussy willow (16). Large dark raisin is heart cleanser of bad humor and is a heart booster (24). Cooking sugar beet in hot ashes, peeling it off, sliding it and cover it with sugar candy powder till a sweet juice is extracted from it, then consume it few times a day and for few days (16). Lemon balm also is tonic for heart and useful for palpitation (24). Table 1 shows the names and characteristics of some useful herbs for palpitation from Avicenna’s Cannon and Razi’s Alhavi (15).

**Table 1.** Names and Characteristics of Some Useful Herbs for Palpitation from Avicenna’s Cannon and Razi’s Alhavi

Name of Herb	Natural Temperament	In Cannon(Quanon)	In Alhavi
**Myrtus**	Cold and dry	Tonic for heart	Useful for palpitation
**Usnea**	Hot and dry	Useful in palpitation	-
***Emblic myrobalan***	Cold and dry	Tonic for heart	-
***Cuscuta epithymum mur***	Hot and dry	Remove the melancholic diseases	-
**Lavender**	Hot and dry	Refines the phlegm and melancholy	-
***Citrus deliciosa***	Skin touch is hot and dry but its inner part is cold and wet	Sourness of citrus is good for hot palpitation but harm for lung	The sourness of its inner part is useful for palpitation
**Prunus**	Cold and wet	Decreases the inflammation of the heart	-
***Melissa officinalis***	Hot and dry	Exhilaratives and removes palpitation	Tonic for heart , exhilarative and remove palpitation
***Ocsium basilicum***	Cold and dry	Seriously tonic for heart	-
***Terminalia chebula***	Cold and dry	Useful in palpitation and panic	Black one is useful for palpitation
***Common chicory***	cold	Poultice it with barley powder on the chest wall is useful for palpitation	-
***Cuecuma zedoaria***	More hot and dry than other herbs	Tonic for the heart	-
***Crocus satyivus***	-	-	-
**(Ssafron)**	Hot and dry	Tonic for heart	-
**Tabasheer**	Cold and dry	Tonic for heart and useful in hot palpitation and syncope due to bile	Is good for palpitation
**camphor**	Cold and dry	It uses for heart treatment	-
**Amber boa**	Hot and a little dry	With cold water is useful for palpitation	Useful for palpitation
***Moshus morchiperus*** ** (musk)**	Hot and dry	Tonic for heart, exhilarative and useful for panic and palpitation	Tonic for heart
***Valerian officinalis***	Hot and dry	Useful in all palpitations	-

## 5. Discussion

In traditional medicine, Palpitation or khafaghan has many causes as in modern medicine but in different wording. Based on its causations, symptoms and signs would be different too, and we can compare some signs of palpitation in traditional medicine to modern medicine, for example one is: hot palpitation may be compared with fast cardiac beats dues to increasing heart rate. In cold predominance of body or heart, the apparent symptoms in the pulse or in whole body are totally different when hot temperament is predominant. For instance in dry-cold predominant over the heart or whole body, the patient shows panic and fear symptoms. Perhaps we can compare the palpitation in panic attacks in modern medicine with melancholic palpitation in traditional medicine. Further studies and the knowledge of common diseases that contribute to palpitation or heart diseases in general might show better ways for treatment of heart diseases.

At the end, we can pay attention to the attached table of single herbal medicines, which Avicenna and Razi have had in their books. They are useful for the treatment of palpitation according to both scientists. More authenticating documents need to do clinical trials to improve their recommending drugs for palpitation treatment. In this article we just explained the herbs they used. This article as every other one has strong and weak points. Strong point is detecting another view which is related to Iranian traditional medicine. This aspect is completely different from modern medicine.

As we know traditional medicine looks at diseases based on temperament and looks at every patient holistically very different from modern medicine. By using this aspect we can diagnose and treat patients and avoiding modern medical drug side effects. The weak point is:

As some useful herbal drugs have been shown here, based on ITM, the main idea was to describe them inessential point, but further researches are needed to detect them in modern medicine and any side effects they may have.

Otherwise in this article only description of diagnose and causes of palpitation in both traditional and modern medicine was the main and grail aim, herbal drugs are only accessory. 

This study is largely of exploratory nature. The findings of this study are useful for palpitation diagnosis based on traditional medicine aspect. This study suggests some herbal drugs to be useful for palpitation treatment. Some single herbal therapy for treatment of this disease was extracted based on two famous traditional books, one book is named Alhaavi of Razes and the other one is Al-Qanun Fi Al-Tibb" or "The Canon of Medicine of Avicenna. But we hope in future we can do clinical trial on each of herbs which were recommended long years ago.
